# Shift Happens: Emergency Physician Perspectives on Fatigue and Shift Work

**DOI:** 10.3390/clockssleep5020019

**Published:** 2023-04-18

**Authors:** Zachary Klinefelter, Emily L. Hirsh, Thomas W. Britt, Caroline L. George, Margaret Sulzbach, Lauren A. Fowler

**Affiliations:** 1 Aptima, Inc., Woburn, MA 01801, USA; 2 Prisma Health, Greenville, SC 29605, USA; 3 Department of Psychology, Clemson University, Clemson, SC 29634, USA; 4 School of Medicine Greenville, University of South Carolina, Greenville, SC 29605, USA; 5 Department of Physiology and Pharmacology, Wake Forest School of Medicine, Charlotte, NC 28203, USA

**Keywords:** shiftwork emergency physician, fatigue

## Abstract

Research has shown that shiftworkers experience poor sleep and high levels of fatigue. Although considerable research has been performed on fatigue within many shift-work occupations, very little has been done with emergency physicians (EPs). This qualitative study was conducted with the goal of gaining insight into EPs’ perceptions of fatigue at work. Twenty EPs from an academic medical center participated in virtual interviews, with nine open-ended questions asked in a semi-structured interview format. Twelve common topics with four main themes emerged from the interviews. Three of these common themes included sources of fatigue (including both work- and home-related sources), consequences of fatigue (including impacts on individuals and performance), and prevention and mitigation strategies to cope with fatigue. The fourth main theme was the belief in the inevitability of fatigue due to high cognitive load, emotionally taxing work experiences, work unpredictability, and the 24/7 shift-work nature of emergency medicine. EPs’ experiences with fatigue are consistent with but extend those of other types of shiftworkers. Our findings suggest that EPs tend to incorporate the inevitability of fatigue at work into their identity as EPs and experience a sense of learned helplessness as a result, suggesting areas for future interventions.

## 1. Introduction

Emergency physicians (EPs) are responsible for providing medical care to patients in facilities that operate 24 h per day. The day-to-day responsibilities of EPs range from administrative tasks to providing life-saving medical care to ill and injured patients. The nature of emergency medicine (EM) is that the work is unpredictable, and it continues at all hours. This unique combination of job characteristics means that EPs are exposed to the documented stressors associated with shiftwork [1] in addition to unpredictable work tasks and conditions that can include traumatic or even harmful situations. Unsurprisingly, research has shown that EPs experience negative work outcomes, such as fatigue, burnout, turnover, and others, at higher rates than many other professions and medical specialties [2,3]. Research is also beginning to show that the experiences of EPs are only worsening during the COVID-19 pandemic [4,5].

Given these unique circumstances and the importance of ensuring the health and well-being of our emergency care workforce, more research needs to be conducted to better understand how to help EPs. The current study aimed to contribute to the literature base on fatigue in EM by exploring the perceptions and experiences related to fatigue from working EPs. This study’s goals were to understand perceptions of fatigue among EPs working in an academic Department of Emergency Medicine within a hybrid academic/community hospital system, develop an understanding of how EPs experience and deal with fatigue during and outside of work, and provide foundational knowledge for researchers and practitioners to better understand what is needed to help EPs cope with and prevent their fatigue.

### 1.1. Fatigue at Work

For decades, researchers have documented increasing work demands on employees [6]. One source of these increasing demands stems from organizations increasingly shifting toward flexible, 24/7 operations. Studies have shown that more and more employees are experiencing longer work hours, shorter recovery periods, and more unpredictability in terms of scheduling [7]. Organizational research has shown that these changes in work schedules have been accompanied by increases in employee fatigue, burnout, turnover, and other negative outcomes [8]. In addition, research on shiftwork over the past century has shown that shiftworkers experience higher levels of fatigue due to the non-circadian nature of their schedules [1]. Fatigue is defined as a state of displeasure that includes exhaustion or tiredness at a level that affects individuals’ abilities to function normally [9]. Fatigue at work is associated with many negative outcomes, including decreased work performance and decreased cognitive functioning [10]. Within the medical field, fatigue among healthcare providers has also been associated with negative outcomes such as medical errors and other safety incidents [11]. The issue of fatigue in healthcare providers is important because it is directly linked to provider well-being, patient outcomes, and organizational outcomes such as decreased productivity and increased turnover [12].

### 1.2. Emergency Medicine Is Unique

Emergency medicine differs from other physician specialties due to several key factors. The emergency department (ED) is often the first point of entry for many patients within a medical center; therefore, EPs are usually the first point of contact with patients. Many times, these interactions involve emergency situations involving critical illness and injuries. These patient visits are unscheduled, differ significantly in their complexities and needs, involve high-risk decision-making, and occur concurrently, and, therefore, they require the full cognitive capacities of the Eps involved.

Additionally, the EP shift workload is unpredictable and can fluctuate vastly from shift to shift depending on patient arrival times, numbers, and acuity [13]. ED volumes continue to increase yearly and frequently exceed ED capacity. Therefore, shifts in which physicians work at a relaxed pace are increasingly rare. Unlike office-based physicians, EPs have no control over patient arrivals or flow, when they see which patient, and when they may have to pause the care of one patient to attend to a new patient in more dire need. Furthermore, interruptions and changes-of-task in order to manage higher-priority or higher-acuity problems or patients are frequent in the ED environment [14]. Therefore, it is common for EPs to work at their maximal physical, cognitive, and emotional capabilities throughout the duration of a shift.

Another notable difference in emergency medicine is how the shifts are scheduled. EDs are open 24 h per day, requiring constant staffing. EPs also often work a wide variety of shifts, without a particular rotation. This differs from office-based physician specialties, in which the physician works primarily during the daytime. It also differs from surgical and other hospital-based specialties, in which a physician may work during one day, be “on call” overnight, and then may or may not work again the following day. While surgical work hours may be long and fatiguing in their own right, their schedules generally involve less circadian disruption than irregularly scheduled EP shifts [13]. EP shift schedules also differ from those of other hospital-based shiftwork physicians, such as hospital medicine physicians, and also differ from nursing schedules, in that a hospitalist physician or nurse is often designated to work a particular “day” or “night” shift for the majority of their scheduled shifts. Finally, different emergency department (ED) schedules may require shifts of different durations on different days (often between 8 and 12 h in duration).

The unique combination of unscheduled patient arrivals, high patient volumes, high-acuity injuries and illnesses, management of multiple patients concurrently, frequent task interruptions, and the presence of high-risk decision-making, all produce a significant cognitive load in EPs; all this, combined with unpredictable shift schedules, can lead to deeper levels of fatigue in EPs.

Given these unique characteristics, additional research specific to EM is required to understand how fatigue is experienced and to better enhance provider well-being. This need for additional research has become especially salient given the COVID-19 pandemic, as the front-line status of EPs has led to increased patient loads, overcrowding in emergency departments, increased exposure to death and dying, increased risk of infection, and likely many additional fatiguing work factors [15,16]. Bérastégui and colleagues [16] also noted how the characteristics of shifts in EM can contribute to fatigue, including the lack of consistency in shifts, their length, and the uncertainty of having to come in for work when on call. In the present study, we expected EPs to highlight these sources of fatigue. However, we also wanted to determine whether there are additional factors contributing to fatigue that have not been systematically examined [17].

### 1.3. Fatigue and Fatigue Interventions in Emergency Medicine

In a meta-analysis of research on fatigue in physicians, Gates and colleagues [12] showed that, while the link between fatigue and physician well-being is clear, studies have yet to provide sufficient evidence that fatigue leads to negative effects on performance and patient-related outcomes. The authors noted that much of the research linking fatigue with detrimental patient and performance outcomes is theoretical or conducted in controlled lab settings that have weak ecological validity within actual healthcare settings. Although research on emergency medical service (EMS) workers has shown relationships between self-reports of fatigue and reported injuries and errors [17], the nature of the work performed by EMS workers is fundamentally different from EPs in that EMS workers care for a single patient at a time, whereas EPs care for multiple patients concurrently. Best practices for mitigating fatigue emphasize understanding the consequences of fatigue for occupationally specific work outcomes [18,19].

Some researchers [16] have suggested that the reason fatigue does not appear to negatively affect patient outcomes may be due to physicians’ ability to compensate for fatigue-related impairments in their functioning through fatigue-proofing strategies. These strategies are informal fatigue management techniques that include double-checking work, having colleagues review medical decisions, and focusing on one task at a time. This research suggests that physicians can overcome the effects of fatigue, at least in terms of negative patient outcomes, by implementing these informal techniques. However, the researchers have pointed out that these fatigue-proofing strategies can be problematic. For example, these strategies do nothing to provide for the effects of fatigue on the physician’s own well-being. Additionally, evidence suggests that some of these fatigue-proofing strategies are associated with increases in burnout, showing that physicians trade their own well-being in an attempt to ensure patient safety and high-quality care [12,16].

While fatigue intervention research on EM has yielded mixed results, some promising interventions, both formal and informal, include naps during night shifts, having colleagues review medical decisions, and focusing on one task at a time [2,20,21]. In their review of sleep issues and their implications for emergency care providers, researchers [22] have noted that, while certain strategies have shown some ability to reduce fatigue during EP shifts, the most effective strategies involve a combination of efforts from the physicians themselves (e.g., sleep hygiene, diet), institutional factors such as scheduling characteristics (e.g., not switching between night and day shifts too frequently), and on-shift interventions such as brief napping and caffeine usage. The authors also highlighted the notable lack of fatigue risk management systems when comparing EM to other industries such as aviation.

Martin-Gill et al. (2018) proposed recommendations to reduce EP fatigue, some of which included providing EPs with instruments to track fatigue, working in shifts shorter than 24 h, always having access to caffeine on shift, having the ability to nap on duty, and receiving educational training on fatigue-reduction techniques [23]. Working shorter shifts has been shown to reduce fatigue [24]. However, some hospital systems do not have napping rooms, and even if on-call rooms are supplied, many EPs are simply not able to sleep on shift due to patient needs and volume. Additionally, even if caffeine products are offered to Eps on shift, many Eps have reported that they either do not have time to eat a snack, or even to hydrate, while on shift or that if they do take a short break to eat, they feel guilty for doing so [25]. Additionally, there is a misconception that EPs are not allowed to eat or drink at their workstations, which may act as an additional barrier to doing so (however, the Joint Commission released a statement disputing this myth) [26]. Lastly, while education on fatigue may be helpful, if the environment does not change, training will likely be futile.

As mentioned earlier, there is a lack of empirical studies examining physician fatigue in EM, and few qualitative studies have been conducted examining physician experiences related to fatigue. Berastegui and colleagues conducted a focus group study to address how fatigue operates in EPs, showing that many physicians report experiences consistent with the literature on fatigue in other settings [16]. Additionally, this study showed that physicians employ a wide range of strategies in preventing and dealing with fatigue at work. Finally, their study showed that EPs might benefit from the implementation of more formal programs that would help physicians learn other strategies, which would also make organizational leaders aware of what EPs need to do to address fatigue on a day-to-day basis. Although their study addressed how fatigue is perceived to influence performance and EPs’ own attempts to reduce the effects of fatigue, the study did not address sources of fatigue or broader recommendations for reducing the negative effects of fatigue.

### 1.4. Current Study

The management of fatigue in other industries has been addressed with the implementation of fatigue risk management systems, which have become popular in aviation, transportation, and other mission-critical industries [27]. Fatigue risk management systems require an assessment of the current state of fatigue within the existing industry in order to identify possible areas of improvement [18]. Ideally, this research on existing fatigue would show not only objective assessments of fatigue but also perceptions of fatigue from those being studied. Although some prior qualitative research has begun to address the unique experiences of fatigue in EPs, there remains a need to better understand physician perspectives of fatigue as an issue and their recommendations for addressing fatigue in emergency medicine settings. In other words, to what extent do physicians feel fatigue is an issue in EM, and to what extent do they feel it is an individual versus institutional responsibility to intervene? Understanding the answers to these questions is critical to developing effective long-term interventions for fatigue in EM. Given the gaps in the knowledge base noted previously and the need to better understand specific perceptions of EPs, the current study employed a qualitative approach to elicit the experiences and perspectives of EPs related to fatigue in their work. We approached this study with the following research questions in mind: What do EPs feel are the current state and sources of fatigue in emergency medicine (RQ1)? Do EPs experience fatigue in terms of sources and consequences, in a manner consistent with the literature (RQ2)? What do EPs perceive as the most promising path forward in terms of reducing fatigue (RQ3)?

## 2. Results

The characteristics of study participants (*N* = 20) are displayed in Table 1. The age range was from 30 to 63 years, and the mean age was 42.6 years (40.5 median). Fifty-five percent of participants identified as female, and forty-five percent identified as male. The majority of participants identified as “White” (75%), with 20% identifying as more than one race and 5% identifying as Asian. Time spent practicing EM ranged from 2 to 31 years, with an average of 11.4 years (7.5 years median)

After compiling all interview data, 12 topics were coded. Of those topics, four main areas of emphasis emerged, with a strong thematic presence in the interviews that is pertinent to the research questions in this study: the inevitability of fatigue in EM, sources of fatigue, consequences of fatigue, and prevention and mitigation strategies. Within each of these four areas, themes or codes are presented below, with selected comments from participants listed in corresponding tables. Sources and consequences of fatigue are further divided into the work and home contexts. Two numbers are presented with each code; the first represents, out of seventeen, the number of interviews/focus groups in which the code was discussed (fifteen individual interviews and two focus groups, with two and three participants, respectively); the second number displays the number of times the code was discussed across all participants.

### 2.1. The Inevitability of Fatigue in Emergency Medicine (Table 2)

Many of the participants described how being an EP is shiftwork and that, in shiftwork, fatigue is common. However, many participants went further, describing fatigue in EM as inevitable. The interviews revealed that many EPs feel that fatigue is inescapable due to the combination of shiftwork and the unique work characteristics of emergency medicine. The code indicative of this theme had to do with fatigue being inherent to being an EP.

**Table 2:** Examples of comments from EPs about the inevitability of fatigue in emergency medicine.

Inevitability of Fatigue *	Comments
Nature of Emergency Medicine (12/17, 28)	“I think fatigue is inevitable in emergency medicine, regardless of where you work. It’s just the nature of the beast. When you have to cover a department that it’s chaos, that literally all hours of the day, we don’t have the luxury of closing the emergency department during inconvenient hours. And you can’t get enough people to work the horrible shifts all the time” “Emergency Medicine is shift work, that’s just the reality and there’s no Emergency Medicine job that’s not shift work. And it’s very rare to find a place where you can just go and say, I only want to work day shifts on these three weekdays and do it.” “Some of the things are just inherent in properties of our job, like it or not. It’s not going to be structured. It’s not going to be one patient at a time, when I’m done with that I can move to the next one. So, I mean, there are going to be interruptions.” “And some people would say, “Well, who cares? You guys don’t work every day, Monday through Friday.” But I think because of the nature of emergency medicine where we don’t have scheduled times, we can have a whole bunch of patients at once, we can have whatever, and then the fact that you can work eight, 10, 12 h and you don’t really have any sort of scheduled... We don’t have lunch hour, right?” “ER medicine has a pretty high burnout rate, and compared to some other specialties. And I think it’s because people start getting to a point where they’re like, “Man, I’m done with the whole all over the place, it’s randomized. I don’t know if I’m working or not, and when I do work, everyone else is off.” “I think most people would have a little less trouble with being fatigued if they have all day shifts, but the department has to run all the time.”

* The numbers presented with each code represent the total number of interviews (out of 17) in which the code was discussed, with the second number indicating the number of times the code was discussed across all participants.

#### Nature of Emergency Medicine (12/17, 28)

As noted in Table 2, participants shared that many of the sources and consequences of fatigue in their work feel inevitable. Fatigue being an inevitable part of being an EP was the most frequently mentioned code in the entire study. The participants seemed to suggest that fatigue is inevitable for two main reasons: first, because emergency medicine involves shiftwork and, second, because of the unique nature of the work. As one example, having to deal with serious, life-threatening situations with multiple patients concurrently, with little to no time to pause or rest while working, and having to do so in an unpredictable manner, while also dealing with the demands of shiftwork such as irregularly rotating schedules and lack of sleep, will inevitably lead to fatigue.

### 2.2. Sources of Fatigue

Participants described numerous sources of fatigue, including many stemming from work, as well as sources from outside work. We identified 19 codes in the data that represented distinct sources of fatigue discussed in the interviews, with 16 work-related sources and three non-work-related sources. Below, we present the five most discussed work-related sources of fatigue and all three of the non-work sources.

#### 2.2.1. Sources of Fatigue at Work (Table 3)

*Shift pattern* (13/17, 35). This code represented causes of fatigue related to irregular or inconsistent rotations or patterns of shifts. Participants generally mentioned this source of fatigue early and often throughout the interviews, with this source being applied twice as much as the next most prevalent source of fatigue.

*Work volume* (10/17, 17). Next to shift patterns, participants often discussed being fatigued due to the high volume of work during shifts. This volume could be in the form of high numbers of patients, as well as high-acuity patients.

*Blocks of shifts* (10/17, 15). This code describes experiencing fatigue as a result of working multiple shifts without days off in between. Participants talked about the feelings that they experience when they think about an upcoming string of multiple shifts in a row, as well as how they feel during and toward the end of those strings of shifts.

*Fatiguing interactions with others at work* (7/17, 11). For the most part, participants mentioned that fatiguing interactions with others on shift occur in the form of interruptions from coworkers, subordinates, patients, and patient family members. Some participants even mentioned interruptions as the most fatiguing aspect of being an EP.

*Insufficient or malfunctioning equipment* (6/17, 10). EPs discussed how having equipment issues in the ED can be fatiguing. Participants mentioned that these issues can be disruptive, sometimes even leading to the EP having to make calls to have equipment fixed themselves or request replacements. One additional factor that was mentioned related to equipment in the ED was that many of these participants work at multiple ED sites across the healthcare system. Participants talked about how equipment can be different in terms of quality and functionality across sites, which can lead to frustration and even fatigue.

**Table 3:** Examples of comments from EPs about sources of fatigue at work.

Sources of Fatigue	Comments
Shift Pattern (13/17, 35)	“Some of the fatiguing factor is that you can have quick turnarounds where you never have a set sleep wake cycle, which really, evidence is out there that obviously impacts people a lot, if you’re constantly going to bed at one night it’s 11:00 PM, the next night it’s 3:00 AM, the next day it’s 7:00 AM, you know?” “If I have a particular month that’s really heavy on nights that aren’t really grouped together well, that’s the kind of schedule that’s the hardest for me. If they’re grouped together so that you can develop some consistency over the period of say a few shifts, that’s fine. But if I have five or six night shifts in a month that are all scattered around and not next to each other, I find those months to be a lot harder, personally.”
Work Volume (10/17, 17)	“Sometimes the volume ratio between providers and patients can be fatiguing in terms of how much you’re trying to juggle at one time, which is unique to emergency medicine, because most areas of medicine have scheduled appointments or operating room times and stuff, so you don’t have that kind of influx of patient volume and that kind of stress, which I think fatigues people as much as anything.” “I think if there’s a lack of physician coverage, that can be very fatiguing if you’re finding the departments so busy and the flow is off so that you can’t actually get any of your charting done and they just keep bringing patients back, it can be very overwhelming…”
Blocks of Shifts (10/17, 15)	“I actually will kind of dread the beginning of those stints, but I have two coming up, so tonight is actually my last night before maternity leave, which is very exciting, but I do have two weeks of five in a row coming up with just a day off in between. I will notice that at home, I will start to be less productive; I don’t really want to do the things that I would normally do on a daily basis, because I feel like I have to conserve my energy knowing that I have these shifts coming up. It’s the same if I’m working a string of nights. I feel like I have to conserve my energy. Unfortunately, that also makes it worse, because I want to conserve my energy, so therefore I don’t want to go out and do physical things or be very social, then I can’t sleep because I haven’t been as active during the day. So it all does kind of compound negatively.” “I worked with a resident the other night who it was his fifth night shift in a row. And you could tell by halfway through the night, he was mentally on his way out, just couldn’t take a lot more. And it was my first night, so I was fresh and ready to go.”
Fatiguing Interactions with Others at Work (7/17, 11)	“So the number one thing is interruptions … the ED doc is constantly interrupted, and you could have a nurse asking you for one thing on your left hand side, and then a tech handing you an EKG while you’re on the phone with someone. So you’re getting interrupted. Say you’re on the phone with someone, you can be interrupted like three different times while you’re on the phone. And so that leads to a lot of fatigue.” “Definitely distractions, I think … the more your attention is diverted away from what you’re doing, the more mental energy you’re expending. And I think that’s very fatiguing over the course of a day.”

#### 2.2.2. Sources of Fatigue Outside Work (Table 4)

*Childcare duties* (9/17, 12). The most common source of fatigue outside of work that participants discussed was childcare.

*Insufficient sleep quantity or quality* (9/17, 11). Poor or insufficient sleep was mentioned by participants in nine of the interviews as a source of fatigue.

*Other home duties* (4/17, 5). This code included sources of fatigue related to demands at home aside from childcare. Participants mentioned tasks such as cleaning, as well as just generally needing to address other life issues. For the most part, these demands were described as fatiguing due to reducing the time for relaxing and sleeping between shifts.

**Table 4:** Examples of comments from EPs about sources of fatigue outside work.

Sources of Fatigue	Comments
Childcare Duties (9/17, 12)	“And some people, they have kids, right? So they get home at 7:00 or 8:00 AM, have to take their kid to school and don’t really fall asleep until later. That’s very tough for your sleep hygiene. That obviously affects your work in the ED.” “So that is tough, just having little kids, for sure. Especially because when you get off your shift, you are the primary caregiver. Even if you have other family members at home, the kids just tend to prefer mom to put them into bed and to do everything. Once I’m home, the kids want me to do it. So I don’t really get to rest after a shift. I just kind of start my second job being a mom.”
Insufficient Sleep Quality or Quantity (9/17, 11)	“I think sometimes I ask myself, can you have physician wellness without feeling like you have slept well and that your sleep tank is full? I feel like that’s one of our baseline physiological needs is sleep. And I feel like you can’t even begin to talk about physician wellness until you’ve at least made sure everyone’s getting enough sleep.” “Things that contribute to fatigue, not sleeping well, which can also go along with scheduling, but at the same time, it goes along with being nine months pregnant. It’s harder to get really good sleep at night before shifts, and so I think that that can contribute to fatigue.”
Other Home Duties (4/17, 5)	“And another is just outside of work, that’s just other life issues that need to be addressed, whether that is family or physically just having to address issues, or I should say physically have to be somewhere else, which then decreases your ability to sleep or prepare for your next clinical shift.”

#### 2.2.3. Consequences of Fatigue at Work (Table 5)

*Less compassion toward patients and colleagues* (13/17, 22). Participants noted that one of the early consequences of being fatigued at work is a decrease in their ability to display compassion toward patients and colleagues.

*Decision-making ability suffers* (10/17, 18). Participants also discussed how their ability to make decisions can be inhibited by fatigue.

*Consequences related to the quality of patient care* (7/17, 10). While certain consequences were coded separately, such as decision-making ability, patients also discussed more generally that patient care tends to be at a lower quality when they experience fatigue at work.

**Table 5:** Examples of comments from EPs about consequences of fatigue at work.

Consequences of Fatigue	Comments
Less Compassion Toward Patients and Colleagues (13/17, 22)	“Your compassion decreases the more tired you get. You just lose that empathy because, yeah, you’re just so tired, and it’s tough then to keep going with that.” “I’ve learned through the years, the first thing to go is my sunny disposition. And when I’m fatigued, I think I still make appropriate decisions and good medical management, but I’m not very nice and can be perceived as rude.” “There is more margin for compassion when rested. And a lot of people in the in-patient who come into the emergency department really need a grandmother. And there’s just a lot more space for that expression of caring that helps those people feel better and feel like they have a handle on what’s going on. Especially when it’s something minor at 3:00 or 4:00 AM when the body is just always tired.”
Decision-Making Ability Suffers (10/17, 18)	“It affects decision-making as well. There’s a greater probability of errors when people are fatigued. And I actually think there’s some sort of a tendency towards just flat out being a little bit more rash and blowing off things that otherwise might be an obvious concern.” “I find that I can’t think as quickly, make decisions as decisively sometimes. I’ll find myself being a little more indecisive or taking longer to think through things. I feel like I can’t recall information maybe as quickly or I don’t trust that I’m recalling it.”
Consequences Related to the Quality of Patient Care (7/17, 10)	“I would say, I’m much slower at my job. I see less patients per hour, much less efficient. And certainly, when you’re more tired, like at the end of a shift, you tend to work up patients differently. I’m more aware that I’m tired and then I might not be thinking right, so I’ll order more CAT scans, more blood work, things that may not be necessary for the patient, but to guide me, to make sure I’m not missing something.”

#### 2.2.4. Consequences of Fatigue Outside Work (Table 6)

*Sleep* (10/17, 16). While many participants discussed how sleep issues can be a source of fatigue at work, sleep was also highlighted as a consequence of being fatigued from work.

*Childcare* (5/17, 6). Participants mentioned that their childcare responsibilities often suffer as a result of the fatigue of being an EP.

**Table 6:** Examples of comments from EPs about the consequences of fatigue outside of work.

Consequences of Fatigue	Comments
Sleep (10/17, 16)	“So more of the emotional shifts I think affect my ability to sleep. If I have a really difficult case or a death of a patient, I think that affects my ability to sleep more than the length of time that I’ve worked. Because usually if I work a long shift and I feel fatigued, I think that I can get to sleep pretty well when I get home, just usually not within the hour of coming home. I kind of have to decompress before my energy level has come down enough to allow me to sleep.” “I know a lot of my colleagues, they have trouble sleeping right when they get home. And that’s especially important after a later shift. If you have a 5:00 PM to 1:00 AM shift and you get home at like 2:00 AM, if you can’t fall asleep for an hour or two it’s 3:00 or 4:00, and then you’re waking up mid morning, and that’s very tough for your sleep-wake cycle. So in terms of sleep strategies, the best thing to do is fall asleep right when you get home. And if you can’t do that, it’s tough.”
Childcare (5/17, 6)	“It also makes it then tough on family and kids life at home, which I think adds another different layer to fatigue. Because then it puts a strain on relationships at home because you’re already fatigued, and then it’s just a cascade effect.” “If I’m physically tired [from work], my patience is so much less for my kids and I’m much more excited to just get them in bed so I can actually recuperate physically.”

### 2.3. Prevention and Mitigation of Fatigue (Table 7)

The participants discussed multiple prevention and mitigation strategies throughout the interviews. These ranged from maintaining healthy sleep, exercise, and dietary habits to having strategies in place during each shift in case they were experiencing fatigue at work.

*Sleep* (10/17, 14) To prevent fatigue, participants discussed the importance of maintaining as close to a regular sleep cycle as possible, along with the importance of getting a sufficient amount of sleep before each shift.

*Caffeine* (10/17, 12) Participants spoke of the use of caffeine as a way to mitigate fatigue during shifts in the ED. Some even described caffeine as one of the only options, as it does not require taking longer breaks and can be consumed while working.

*Pace* (9/17, 12) As a strategy to mitigate fatigue, participants described how, when possible, they purposely slow the rate at which they acquire patients, especially toward the end of shifts.

*Exercise* (10/17, 10) In terms of mitigating fatigue during shifts in the ED, participants discussed the importance of getting small amounts of exercise, such as a brief walk outside or some light stretching. Some participants noted that this strategy is not always possible, but when it is, exercising during a shift can help mitigate fatigue during shifts.

*Eating* (9/17, 10) Participants described eating small snacks, sugar, or even full meals during shifts as a method to mitigate fatigue. Often, participants mentioned eating in conjunction with other mitigation techniques such as taking a short break, going for a walk, and consuming caffeine.

**Table 7:** Examples of comments from EPs about prevention and mitigation of fatigue.

Prevention and Mitigation Strategies	Comments
Sleep (10/17, 14)	“… to have respect for the amount of sleep that you get. My husband and I were actually talking about that today, how we like that we respect each other’s sleep, that it’s not a luxury to get eight hours of sleep. It’s an absolute necessity for health. It’s like eating the right food every day and working out. Sleep is right up there for us. So I think that would be one thing.” “Definitely looking ahead at your sleep schedule, try and anchor some time. If I’m doing an overnight shift, I will take a nap before that shift. I have to transition then to meetings for the next day. I will definitely try and get back on a daylight circadian rhythm again so I will sleep, but not sleep all day, get myself up.”
Caffeine (10/17, 12)	“There’s really not much I can do, except for try to force myself to take naps. And then I’m relying a little bit more on caffeine by the third and fourth [shift in a row]. So I guess I try to implement a sleep strategy, and increase my caffeine consumption.” “Caffeine, sugar. There’s not really time to walk away and take a break. Yeah. It’s those two things, I would say, mostly.”
Pace (9/17, 12)	“There are times where if you don’t check yourself, you may have too much on your plate at one time, but you don’t have to, if you don’t want to. You can kind of scale it back so you have that flexibility to help reduce your own shift stress by limiting how much you have on your plate at one time.” “… at about 4:00 AM, I’m going to feel like I’m hitting the wall, and just being cognizant of that. And if I need to take more time to look at things or dig a little bit deeper, then I need to do that.”
Exercise (10/17, 10)	“You have to try to go get a breath of fresh air, go get a drink of water, go get something to eat, and try to make time for it. That’s what I do sometimes. I’ll intentionally go for five minutes and just walk outside. I don’t even do anything. I just walk outside for a minute, just to clear my head a little better, get some fresh air.” “I try to get up and walk. If I notice I’m just feeling sluggish and not into it and I’m just almost wishing I was somewhere else because I’m so tired and I don’t feel like I can get through the shift, I can get up and walk or I go to the physician’s lounge …”
Eating (9/10, 10)	“I get tired if I don’t get a chance to eat or hydrate. So if I’m on shift and I’m feeling funky then I’ll look and say, okay, how am I doing in terms of like basic body functions? Am I eating?”

## 3. Discussion

This qualitative study provides clear evidence that fatigue is a common and prevalent issue for emergency medicine physicians. EPs’ experiences with fatigue regarding its sources and consequences are consistent with other types of shiftworkers and also provide new insights. The common themes expressed across the interviews and focus groups revealed that there are many similarities in the experiences of fatigue across EPs, but there was not one theme common to every participant. However, the most common theme expressed was the inevitability of fatigue in EM. The EPs appear to be approaching the idea of fatigue with a sense of learned helplessness. The idea of learned helplessness is that when individuals feel as if they have no control over their environment and cannot escape the aversive nature of that environment they give in and accept the inevitability of what is to come [28]. This can lead to decreased performance and increased burnout and depression at the individual level, but it can also impact organizational outcomes [29].

The perception that fatigue is inevitable in EM likely comes from beliefs regarding the uncontrollability of various sources of fatigue. Many sources of fatigue mentioned by EPs in this study were consistent with determinants reported in prior research, such as shiftwork, the pace of work, and the unpredictability of the workday [2,5]. However, EPs in this study mentioned additional sources of fatigue not examined in prior research, including interactions with fellow healthcare personnel and patients and their families, as well as sources of fatigue from outside of work, including insufficient sleep, childcare, and other home duties. Although prior research examining the work–home interface has been conducted outside of EM, [30] future research should examine how the demands of EM affect physicians’ non-work lives and how home dynamics can affect EPs during their shifts.

The results of the present study replicated prior research examining the strategies EPs utilize to prevent fatigue from having negative effects on the quality of patient care [16]. Physicians in this study emphasized getting adequate sleep, taking caffeine, slowing the pace of work, eating snacks, and getting brief exercise when possible. Interestingly, although physicians were asked about things the emergency department could do to reduce fatigue, rather than identifying a set of recommendations, EPs were more likely to further discuss the inevitability of fatigue, again demonstrating their sense of learned helplessness.

It is our plan to use the results of the present study to inform the development of a Fatigue Risk Management System (FRMS) specifically for EPs. Sprajcer et al. (2022) recently conducted the first comprehensive review of FRMSs and found evidence for the effectiveness of FRMSs in certain circumstances. Even though the review found overall evidence that FRMSs can be significantly impactful in reducing fatigue, certain FRMSs lack evidence of their effectiveness. Sprajcer et al. also noted that only a small percentage of the studies purporting to assess an FRMS actually analyzed the effectiveness of the FRMS (which includes some assessment of the risk and perceptions of fatigue prior to the study) and went beyond solely describing ideas for fatigue mitigation techniques [27].

FRMSs have mainly been developed for occupations outside of healthcare that are prone to fatigue, including firefighters and other first responders, pilots, and truck drivers [27]. However, as discussed in the introduction, many elements of traditional FRMS programs are not suitable for EPs, given their inability to take breaks during their shifts, lack of ability to nap, and the overall volume and unpredictability of their work environment [18]. The findings of the present study, including the belief that fatigue is inevitable and that recovery from shiftwork is insufficient, suggest the importance of system-level interventions to reduce the fatigue of EPs. The main theme of the home environment as a source of fatigue in EM also suggests the importance of an FRMS considering the quality of the work–home interface of EPs as an important contributor to fatigue.

Addressing fatigue involves addressing both individual and workplace contributors to fatigue. While the general principles of fatigue risk management can be applied across many different industries, allowing us to build upon previous research about FRMSs, studies show the importance of considering individual and task-specific differences that contribute to fatigue [18,19,31,32]. Thus, it is vital to consider the occupationally specific tasks of EPs in the development of an FRMS for EM. Although prior research has identified recommendations for components of fatigue risk interventions in general, we believe the findings of this study suggest the importance of physician perceptions, the EM work environment, and feelings of learned helplessness as unique considerations for future research.

### Limitations

Several limitations exist for this study. First, the nature of EM creates a scenario that makes it difficult to schedule focus groups for several people at once. Initially, the study aimed to conduct focus groups for all sessions, but scheduling became prohibitive due to the nature of EM. Therefore, two sessions took place as focus groups, while the others occurred as individual interview sessions [20]. This may have altered the results, as in the focus groups, individuals tended to react and respond to others’ comments.

Additionally, the interviews and focus groups were conducted virtually due to the fact that COVID-19 protocols were in place. The health system in which the employees worked recommended against meeting in person during the time period of data collection.

Finally, the fact that data collection took place during the COVID-19 pandemic may have impacted perceptions of fatigue in the EPs. More research should examine perceptions of fatigue and mitigation strategies used when there is not a global pandemic underway.

## 4. Materials and Methods

### 4.1. Participants

Participants in this study were recruited via an email sent to 120 attending physicians working in the Department of Emergency Medicine (DEM) of a large academic medical institution in the southern region of the U.S. The recruitment email included a brief description of the project and indicated that participants would receive a USD 5 gift card to a coffee shop as compensation for their participation. The first 20 physicians to respond to the recruitment email indicating a willingness to participate were selected as participants. Initially, we planned to hold virtual focus groups with three to five physicians in each. However, after experiencing some difficulty with scheduling the focus groups, we decided to hold individual interviews with the physicians instead.

### 4.2. Procedures and Materials

This study was approved by the Institutional Review Board at the healthcare organization where the study took place. The interviews consisted of an introductory script, nine questions, and a concluding script (see Appendix A) and lasted between 20 and 50 min. In the introductory script, the researcher thanked the participant for their willingness to participate, briefly described the nature of the research project, informed the participant that the information shared would be kept as confidential as possible, and solicited permission for the interview to be audio-recorded for later transcription and analysis. We also asked participants to indicate how long they had been working in emergency medicine and how long they had been employed by the current Department of Emergency Medicine. The interviews were semi-structured in that they consisted of nine open-ended questions but also allowed for follow-up questions to probe for additional information throughout. We developed the nine questions based on the available literature on fatigue in shiftwork occupations. The questions focused on participants’ perceptions and experiences with fatigue in both their work and home lives. Of note, we asked both about general perceptions related to fatigue in EM and their specific experiences. This allowed us to examine the broad perceptions as well as analyze actual experiences. At the conclusion of the questioning, participants were thanked again for their participation and given information related to receiving their gift card.

### 4.3. Analysis

The recorded audio from the interviews was transcribed by a paid transcription service. The analysis of this data followed the Grounded Theory framework for qualitative analysis [33] and the open coding process [34]. This type of analysis supported the emergence of themes from the data without preconceived conceptualizations from theory or previous research. Furthermore, this approach allowed us to learn about the raw perceptions of fatigue in EM and organize those perceptions based on how prevalent they were across participants. To accomplish this analysis, we also followed the guidelines of Consensual Qualitative Research (CQR) [35]. CQR requires that all decisions related to the coding of data are guided by team consensus, rather than by a single researcher.

The qualitative analysis process involved creating a codebook that comprehensively described the data and then coding the full dataset to determine the prevalence of each of the codes (or themes) in the data [33,34]. To develop the initial codebook from which the full data could be coded, we selected at random 4 of the 20 interview transcripts to be reviewed. The research team reviewed this sample of data individually and developed lists of codes that described the meaningful quotes. We then combined the lists of codes from each researcher into a final codebook, holding group discussions to make decisions about omitting redundant codes. The researchers then reviewed four additional transcripts to ensure that the codebook covered all important information and to demonstrate reliability across researchers.

Once the codebook was created, a researcher used the qualitative analysis software Atlas.ti 8 [36] to review each interview transcript and apply codes to the relevant utterances in each. This process allowed researchers to review the prevalence of each of the codes in the codebook in terms of how many participants discussed each code and in how many interviews each code was discussed. Additionally, the research team was able to further organize the codebook into broader categories, or parent codes, and could then examine the prevalence of the parent codes in the data.

## 5. Conclusions

This study represents one of the first studies to examine the concept of fatigue in EM using a qualitative approach. EPs in this study overwhelmingly confirmed that fatigue is an issue for them in the workplace, and there is a need for effective strategies to cope with this fatigue to be developed. An inability to address the sources of fatigue, as well as an inability to mitigate and counteract the fatigue, leads emergency medicine physicians to feel as if fatigue is inevitable, creating a sense of learned helplessness. More research into the inevitability of fatigue in emergency medicine is necessary to address its impact on both individuals and the hospital system itself.

## Figures and Tables

**Table 1:** Demographics of focus group participants.

Characteristic	Number of Participants (%)
Gender	
Female Male	11 (55) 9 (45)
**Race**	
Asian Two or More Races White	1 (5) 4 (20) 15 (75)
**Characteristic**	**Mean (Median; Range)**
Age	42.6 (40.5; 30–63)
Years in Emergency Medicine	11.4 (7.5; 2–31)

## Data Availability

Not applicable.

## Appendix A

Introductory (script):

Thank you for taking the time to participate in our focus group/interview regarding fatigue management in emergency medicine. The ultimate goal of this project to establish a Fatigue Risk Management System (FRMS) in the ED. This is your chance to make sure your voices are heard.

I will be asking nine questions related to fatigue and fatigue management in the ED. I would like to spend no longer than 5 min responding to each question. That will ensure we conclude the focus group within the hour.

Before we get started, I want to let you know two things. First, the information we learn today will be compiled into a final report. That report will include a compiled summary of all comments made by all participants. This data/report will be shared with the research team in order to drive the development of this program. This summary may be included in a published report of our findings. Secondly, you do not have to answer any questions that you do not feel comfortable with. This interview today is anonymous and confidential. “Anonymous” means that we will not be using your name and you will not be identified as an individual in our report of this project. I would like to record this focus group/interview. The recording will only be used to make sure our notes are correct and will not be heard by anyone outside of this project. Are there any questions or concerns before I begin recording?

The following open-ended questions will be used in the focus groups (directed).

1. What contributes to your fatigue?
2. How does fatigue affect your work performance?
3. How does job-related fatigue affect your sleep?
4. What do you do if you are fatigued?
5. How do you know when you are or one of your coworkers is fatigued?
6. What do you do to prevent fatigue?
7. What is your perception of how your shift scheduling contributes to your fatigue?
8. Do you think you have ever committed a medical error related to fatigue?
9. What could the Department of Emergency Medicine do better regarding physicians and fatigue?

Concluding (script):

- Thank you for participating in this focus group/interview. Your responses will be used when developing a Fatigue Risk Management Program for the Emergency Department. You will receive an email from Starbucks within a few days of participating containing a virtual gift card that you can either print or use with their phone. If for some reason you do not receive the gift card within a few days, please contact Dr. Lauren Fowler at lafowler@wakehealth.edu.
